# Utility of green chemistry for spectrofluorometric and spectrophotometric analysis of vericiguat via reaction with erythrocin B

**DOI:** 10.1186/s13065-025-01485-3

**Published:** 2025-05-06

**Authors:** Hesham Salem, Amany Abdelaziz, Omar Saied, Micheal Amir, Maemona M. Sadik, Noha Khalid, Nadin Mohsen, Dina Z. Mazen

**Affiliations:** Pharmaceutical chemistry department, faculty of pharmacy, Deraya University, New Minia, Egypt

**Keywords:** Vericiguat, Colorimetry, Erythrosin B, Fluorimetry, Quenching

## Abstract

Food colorant Erythrosine B (EB) is an antiviral xanthene dye with a wide range of uses as a colorant in cosmetics and medications. Its availability, affordability, quick labeling, and high sensitivity make it an excellent choice for spectrofluorometric and spectrophotometric examination of amine-based medications. Two quick and accurate spectrophotometric and spectrofluorometric methods were developed for the estimation of vericiguat in this case. To create an ion-pair complex at pH 4 using the Britton Robinson buffer, the suggested methods relied on the interaction between the amino groups of the medication under study and the phenolic group of EB. The quenching effect of the vericigaute drug of EB at excitation/emission wavelengths of 530.0/550.0 nm. This method demonstrated a limit of detection (LOD) of 0.036 µg/mL and a limit of quantification (LOQ) of 0.110 µg/mL, showing rectilinear response in the concentration range of 0.05–0.5 µg/mL. Additionally, the absorbance of the produced ion-pair complex was evaluated using the colorimetric approach at 560 nm, displaying a linearity range of 0.5–10.0 µg/mL with LOD = 0.428 µg/mL and LOQ = 1.298 µg/mL. The greenness of the developed approaches was determined by GAPT and AGREE software for evaluating the suggested methods.

## Introduction

**Fig. 1:**
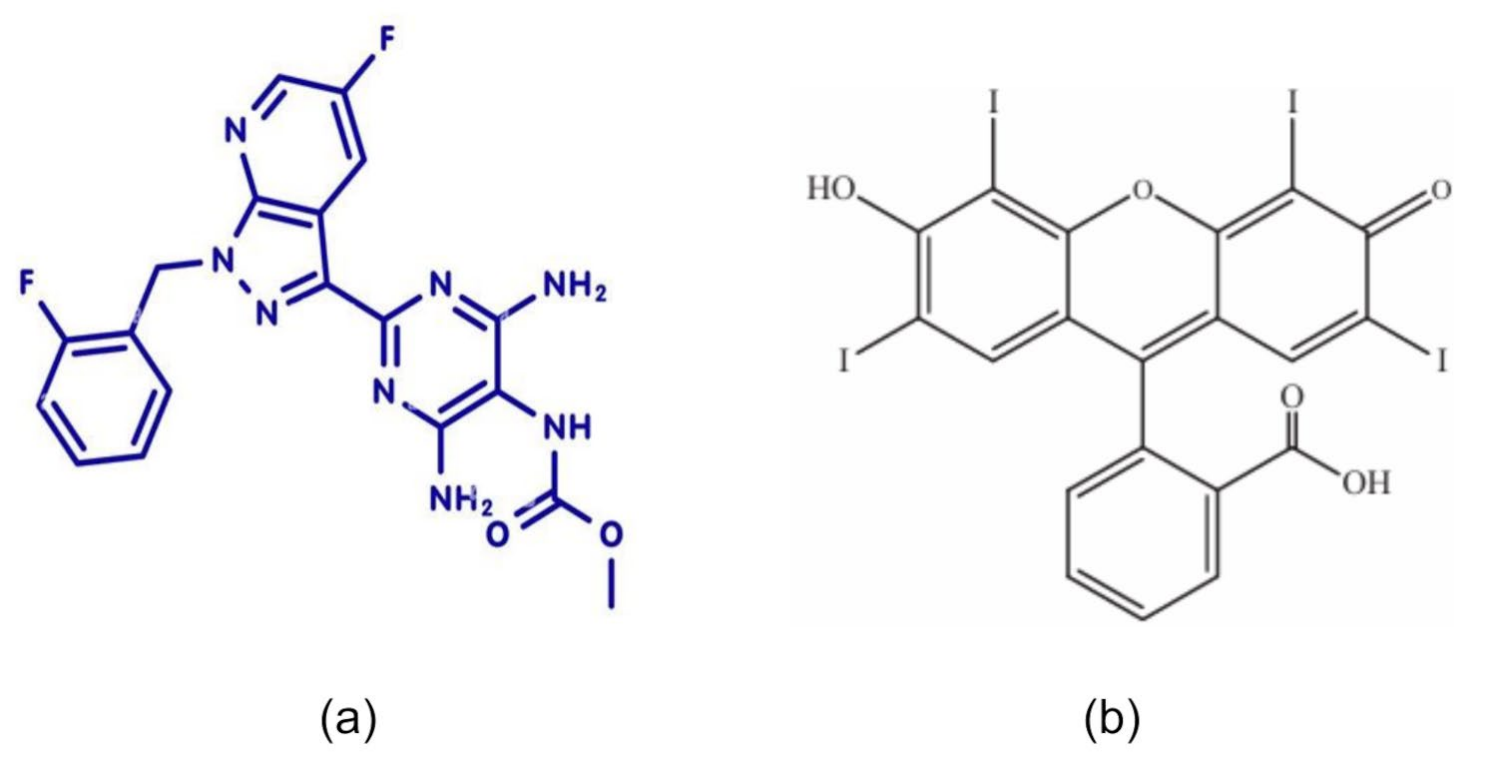
Chemical structure of VER **(a)** and erythrosine B **(b)**

Vericiguat (VER) (methyl N-(4,6-diamino-2-{5-fluoro-1-[(2-fluorophenyl)methyl]-1 H-pyrazolo[3,4-b]pyridin-3-yl}pyrimidin-5-yl) carbamate) (Fig. 1) is an entirely novel soluble guanylate cyclase (sGC) stimulator that is used to treat heart diseases and their symptoms. It processed by two transporters, one of which is called P-glycoprotein and the other called breast cancer resistance protein (BCRP). Vericiguat belongs to medications like riociguat, which was the first therapy in its class to be approved for clinical use in patients with pulmonary arterial hypertension [1, 2]. VER was marketed under the brand name Verquvo ^®^ trademark by Merck since it was given FDA approval and permission for use throughout the European Union in 2021 [3]. Its mechanism of action involves improving cardiac pumping efficiency and relaxing blood vessels, which enhances blood circulation throughout the body. A review of the literature discovered that the drug has been evaluated using a lot of analytical approaches, including chromatographic methods [4–7], spectrophotometric methods [8–10], and spectrofluorometric methods [10]. RP-HPLC method for estimation of the studied drug in bulk and dosage form [4], stability-indicating HPLC/DAD method for quantitation of VER in dosage form and in presence of degradations [5], usage of UPLC-MS/MS for analysis VER [6], investigation of pharmacokinetic interaction of VER with apigenin based on UP-HPLC-MS assay [7], different spectrophotometric methods for determination of the studied drug in presence of its alkaline degradation products [8], utility of diazo coupling reaction for colorimetric analysis of VER [9], and in traduced analysis of Vericiguat via ion-pairing with eosin Y as spectrophotometric and spectrofluorometric probe [10]. To enable a rapid and straightforward evolution of VER with high sensitivity and selectivity, this study introduces a new spectrofluorometric approach in addition to the existing spectrophotometric method. Erythrosine B (EB) (Fig. 1b), a food colorant xanthine dye with antiviral properties against Zika and Dengue flaviviruses [11], is used in a different number of analytical applications, including as a selective and sensitive investigator for the assay for multiple medications due to its ability to adhere to biomaterials and high attenuation coefficients. According to its chemical structure, it is an acidic dye that can form ion-pair complexes with basic amino acids such as VER in a selected Britton-Robinson buffer of pH 4 was observed.

Experimental and theoretical studies of the mechanisms that underlie ion-pair formation, their properties, and applications in various fields have been and still are focused on by researchers since the introduction of the concept in 1926 by Bjerrum. Ion pairs are distinct chemical entities, electrically neutral, formed between ions of opposite charge and held together by Coulomb forces, without formation of a covalent bond. For instance, it has been employed for spectrofluorimetric and spectrophotometric determination of albendazole [12], benzimidazole [13], varenicline [14], trospium chloride [15], tamoxifen [16], sunitinib [17], rupatadine [18], and naftidrofuryl [19].

As a result, in addition to spectrophotometric methodologies, the present study provides the first spectrofluorimetric approaches for quick and simple VER estimation that have excellent selectivity and sensitivity. The two methods were created using green chemistry principles and do not require the use of harmful solvents during extraction. Green Analytical Procedure Index (GAPI) [20] and the Analytical Greenness Calculator (AGREE) [21]. The two green assessment measures were used to examine the environmental benefits of present practices. When this medicine is used, its effect will be observed on both spectroscopic methods. The native fluorescence of EB decreases, which is the basis for the proposed spectrofluorimetric methods; however, the spectrophotometric method showed an increase in absorbance.

## Experimental

### Instrumentation

The spectrofluorimetric measurements were carried out using a JASCO FP-83 spectrofluorimeter. The instrument has a PMT tuned to 400 V and a 150 W Xe-arc light. The excitation and emission monochromators had a slit width of 5 nm and a scanning speed of 1000 nm/min. The spectrophotometric measurements were performed using a T80 twin beam UV-VIS spectrophotometer (PG Instruments, Leicestershire, UK) in conjunction with UV-Win software. The measurements were performed using quartz cells with a diameter of one centimeter. The Aquatron water still 4000d (Cole-Parmer, Staffordshire, UK) was used for creating double-distilled water.

### Materials and reagents

The supplier of the VER standard (purity > 98%) [10] was Bayer Zydus Pharma, Bayer AG Barmen, Germany. VER-labeled Verquvo^®^ 5 mg Tablets were utilized. Merck provided methanol, ethanol, and acetonitrile spectroscopic grades as well as EB (Darmstadt, Germany). Fischer Scientific supplied analytical-grade dimethyl formamide (DMF) from Loughborough, UK. Analytical-grade sodium acetate, ethyl acetate, acetic acid, and acetone were supplied by El Nasr Pharmaceutical Chemical Co. (Cairo, Egypt). Acetic acid, boric acid, and phosphoric acid (0.04 M each) were combined in the proper amounts to create Britton Robinson (BR) buffer solutions (pH: 2.0–6.0). When making the buffer solutions, 0.2 M NaOH was used to alter the pH.

### Preparation of standard solution

The aqueous standard solution of VER (100 µg mL^-1^) was performed by dissolving an appropriate amount of VER (10.0 mg) in 100 mL of distilled water. Further dilutions were then made to obtain the specified range for each method. The reagent EB of two definite concentrations, 1 × 10^-4^ and 5 × 10^-4^ M, was prepared in distilled water for the fluorimetric and colorimetric measurements, respectively.

### General procedure

#### Calibration curves

##### Method I: Spectrofluorimetric method

**Fig. 2:**
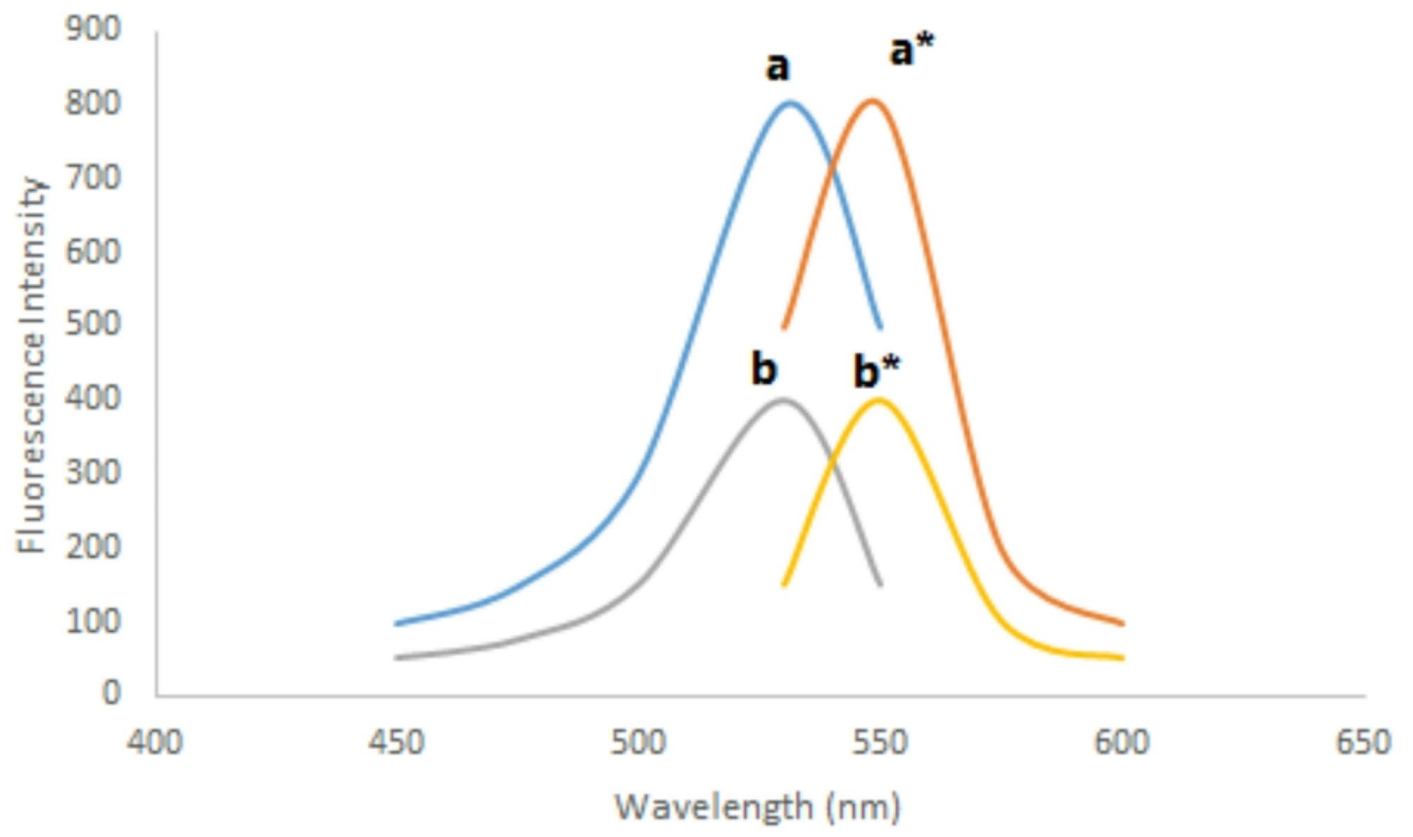
(a, a*) The excitation and emission spectra, respectively, of (1 × 10^− 4^M) erythrosine B (EB) in Britton Robinson (BR) buffer (pH 4) without the addition of the analyte. (b, b*) The excitation and emission spectra, respectively, of the reaction product of (1 × 10^− 4^ M) EB and VER (0.4 µg/mL) in BR buffer (pH 4)

Portions of the standard VER solution (0.05–0.5 µg mL^-1^ ) were translocated in a series of 10-mL glass volumetric flasks. Each flask received (0.5 mL) of BR buffer (pH 4), followed by 0.7 mL of EB solution of concentration 1 × 10^− 4^ M, and then mixed very well. Each flask was filled with distilled water to 10.0 mL. At 550.0 nm, the relative fluorescence intensity was monitored after excitation at 530 nm against a blank experiment (Fig. 2). The quenching in fluorescence intensity (ΔRFI) was then graphed versus the final concentration of the analyte to acquire the corresponding calibration graph from which the regression equation was derived.

##### Method II: Colorimetric method

**Fig. 3:**
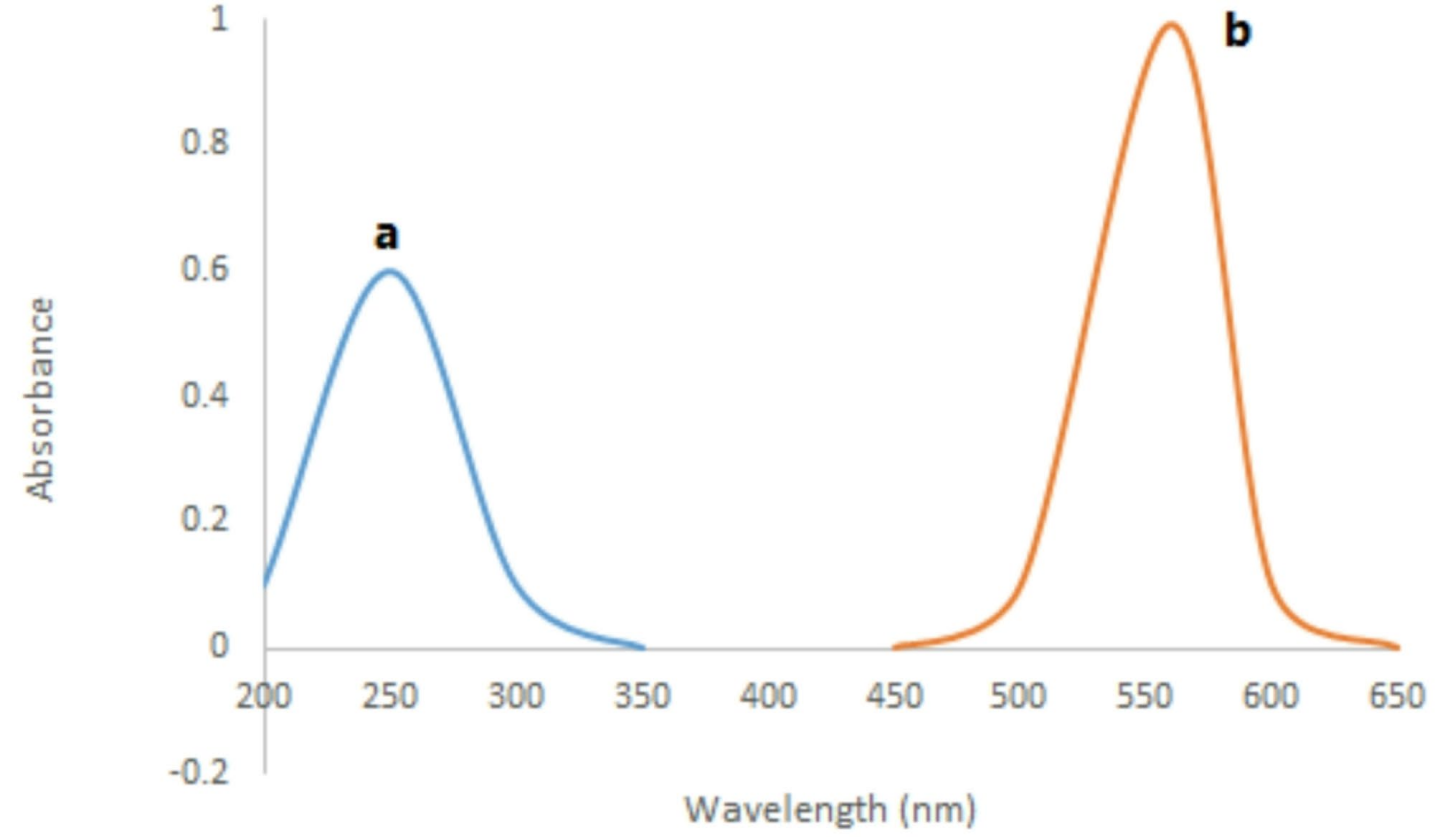
The spectrophotometric absorption spectra of **(a)** VER in distilled water (4.0 µg/mL) and **(b)** the reaction product of VER (4.0 µg/mL) with (5.0 × 10 ^− 4^ M) erythrosine B (EB) at pH 4

The procedure from the spectrofluorimetric method was adapted for the drug within the range 0.5–10.0 µg mL^-1^ using 2.0 mL EB (5.0 × 10^-4^ M). The resultant color was monitored at 560 nm against blank. The absorbance values were graphed against the final concentration of the analyte to acquire the corresponding calibration graph (Fig. 3) from which the regression equation was derived.

#### Assay of VER in tablets

A set quantity of Verquvo^®^ Tablets (10) was weighed and finely crushed. A 100 mL volumetric flask containing 50.0 mL of distilled water and a precisely weighed amount of the powder equal to 10.0 mg VER. The solution was sonicated for 30.0 min, then diluted to one hundred mL with distilled water and filtered. The calibration curve’s main method was followed to generate and assay various quantities. The drug content of the tablets was determined using the appropriate regression models.

#### Determination of reaction stoichiometry

Reaction stoichiometry was determined using Job’s approach and the limiting logarithmic method. Two solutions of VER and EB having the same molarity (1 × 10^-4^ M) were prepared. The general procedure for the spectrofluorimetric method was applied. The values of ΔF were plotted against the mole fraction of VER.

## Results and discussion

In an acidic solution, the acidic dye EB can combine with basic analytes to create ion-pair complexes. When excited at 530 nm at pH 4, the generated compound caused the fluorescence intensity of EB at 550.0 nm to be quenched in the spectrofluorometric method (Fig. 2). The colorimetric method, on the other hand, involves measuring the complex’s absorbance at 560 nm (Fig. 3).

### Optimization of the reaction condition

A number of variables, including buffer pH and volume, reagent (EB) volume, surfactant, dilution media, and reaction time, were examined since they may have an impact on the ion-pair formation between EB and VER.

#### Buffer pH and volume

**Fig. 4:**
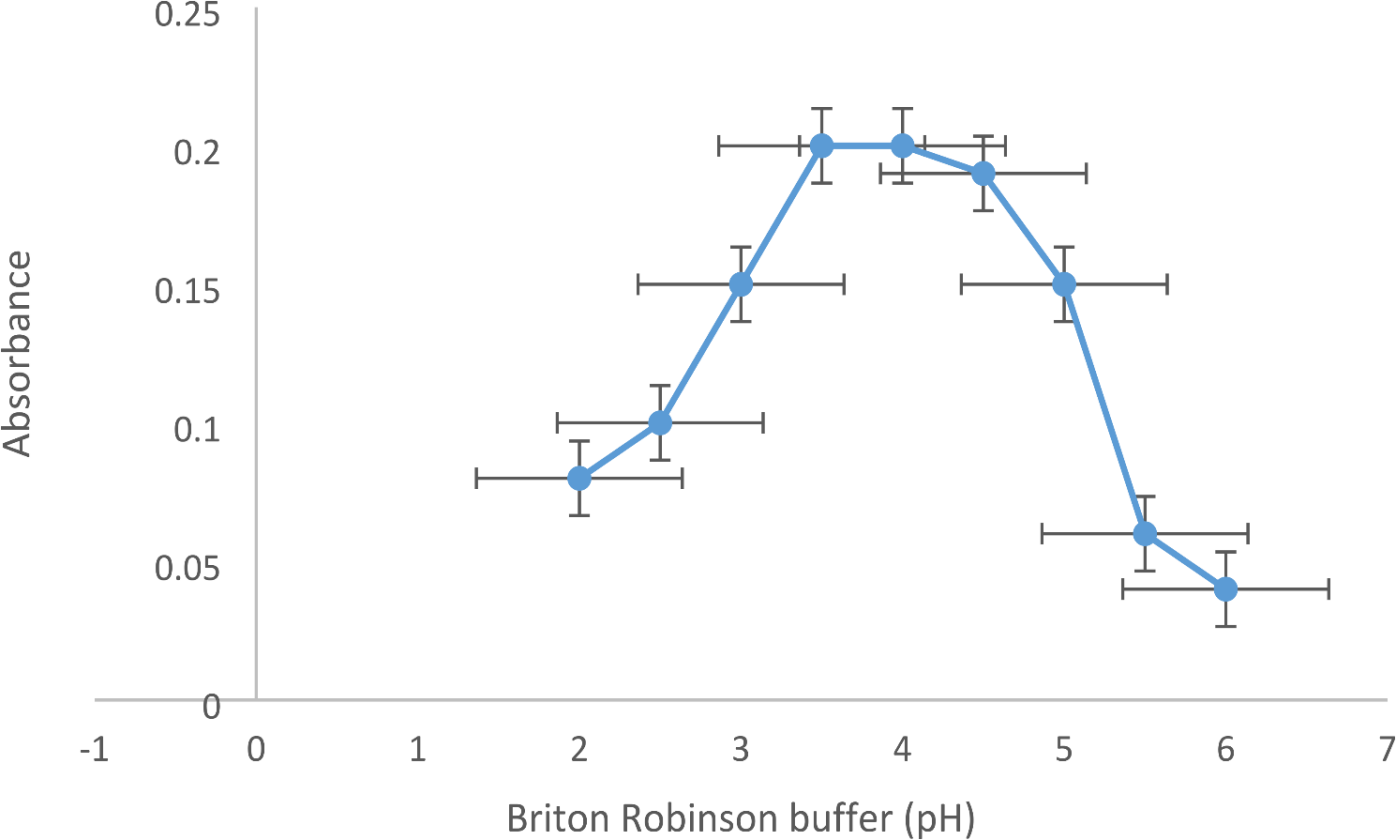
Effect of the pH of Briton Robinson (BR) buffer on the absorbance of VER-EB complex

**Fig. 5:**
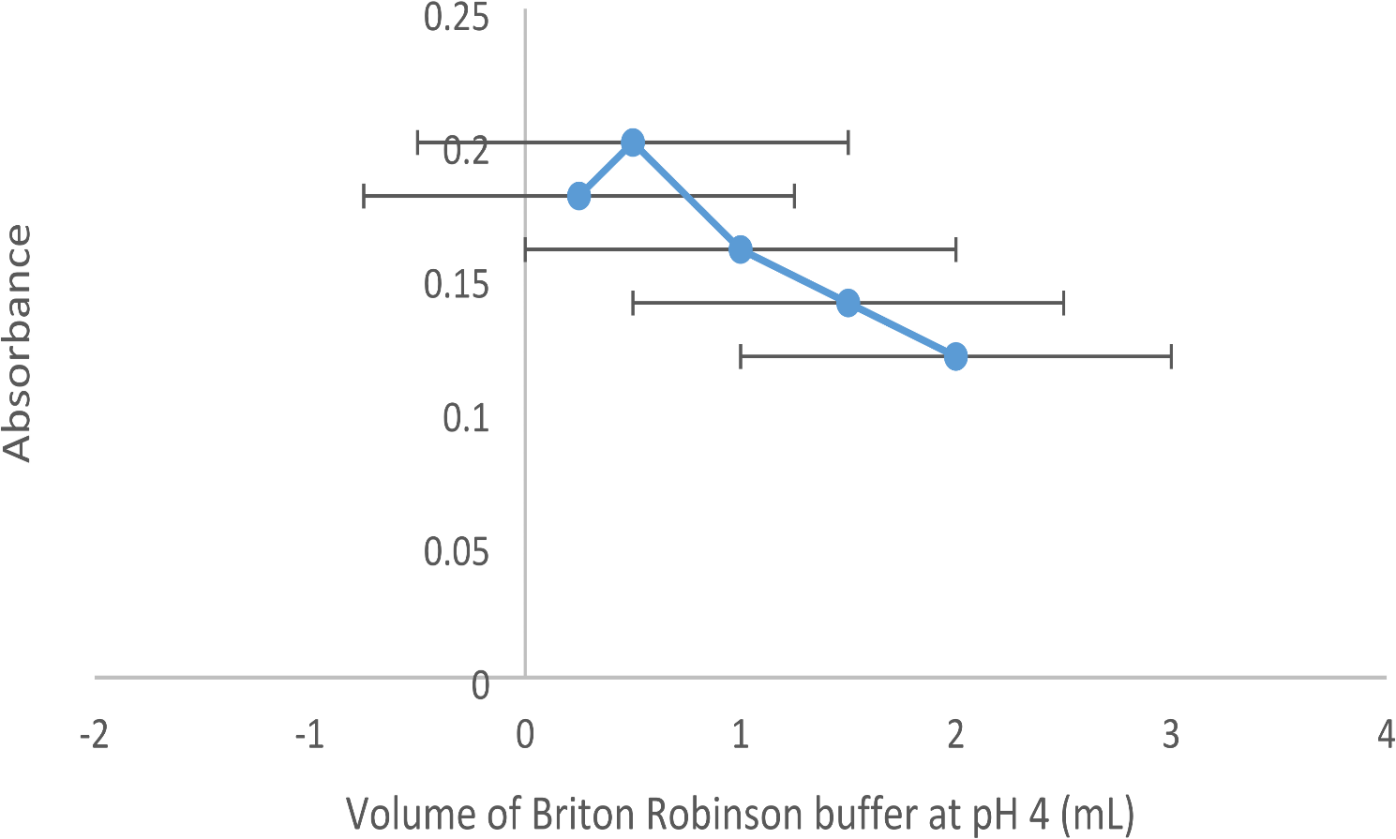
Effect of the volume of Briton Robinson (BR) buffer (pH 4) on the absorbance of VER-EB complex

Varied pH values within the range of 2–6 were studied using BR buffer because EB is an acidic type. It was found that the maximum absorbance was produced at pH 4 (Fig. 4). Most studies of EB quenching by basic drugs proceed in the acidic medium. Different volumes of BR buffer (0.3–2.0 mL) at pH 4 were examined. It was found that 0.5 mL of BR buffer was the optimum volume (Fig. 5).

#### Volume of reagent

**Fig. 6:**
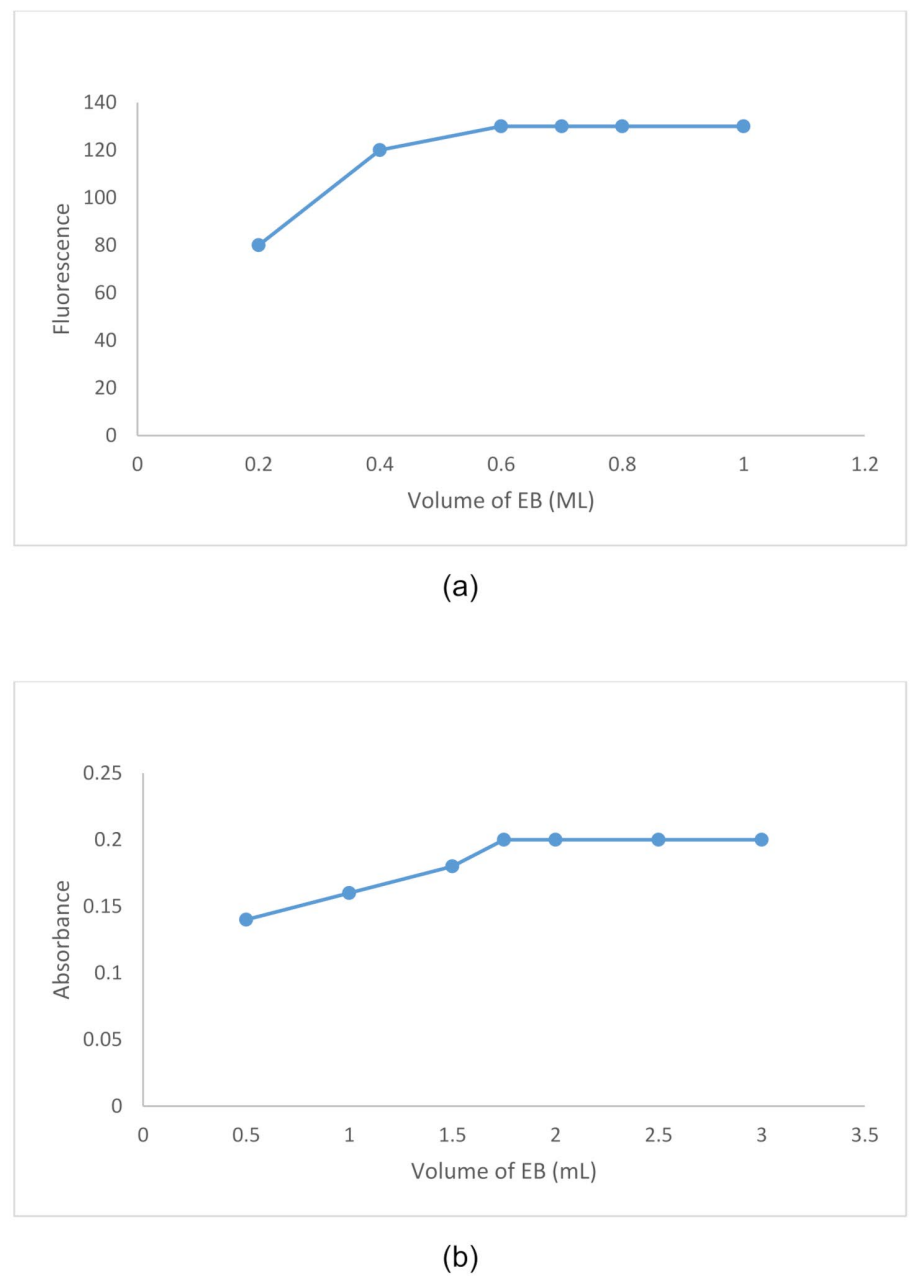
**(a)** Effect of EB volume (1 × 10^− 4^ M) on the fluorescence intensity of VER-EB complex. **(b)** Effect of EB volume (5 × 10^− 4^ M) on the visible absorbance of VER-EB complex

In the spectrofluorometric method, it was found that 0.7 mL of 1 × 10^− 4^ M EB was sufficient to produce the optimum (ΔRFI) (Fig. 6a). For the colorimetric method, 2.0 mL of 5.0 × 10^− 4^ M EB was found to be suitable for yielding the highest absorbance value (Fig. 6b).

#### Reaction medium (diluting solvents)

Ethanol, acetonitrile, methanol, and distilled water were examined as diluting solvents. It was discovered that utilizing distilled water produced the highest absorption, whereas in organic solvents there was no complex formation. The selection of distilled water as a solvent also has many advantages, such as being cheap, available, and green. The EB complex with drugs always shows maximum intensity in distilled water rather than organic solvents, as reported in various studies. Organic solvents may degrade the procedure of the complex’s properties, highlighting and explaining the mild response seen in the study. If particular solvents are used, the complex may get denatured, and the fluorescence value may be changed. Due to their extremely short carbon chains, methanol and ethanol both dissolve readily in aqueous solutions and streamline the procedure by altering the solvent’s characteristics. If more of it enters the system, it could cause a significant disturbance. A very complex system is disrupted by alcohol. As the solvent’s dielectric permittivity logarithm grows, the dominant equilibrium form of EB molar absorptivity rises linearly. Distilled water has the highest dielectric permittivity (ε0 81) and polarity index (10.2) among the aforementioned solvents. The system is entirely miscible since its constituent parts are already dissolved in an aqueous medium. The limited miscibility of other organic diluents, however, may prevent the complex from forming because of variations in their dielectric constants.

#### Surfactants

The probe’s efficiency was evaluated utilizing several surfactants, including cetrimide, SDS, Tween 80, and β-cyclodextrin. All of them were used at concentrations greater than the critical micelle concentration (1.0% w/v). Such surfactants were observed to have no apparent influence on the ion-pair formation, so the work was performed without using any surfactant.

#### Reaction time

Over the course of 45.0 min, the influence of time on complex development was investigated at various intervals. It was observed that the reaction occurs immediately, and absorbance was nearly constant for 60.0 min.

#### Effect of temperature

**Fig. 7:**
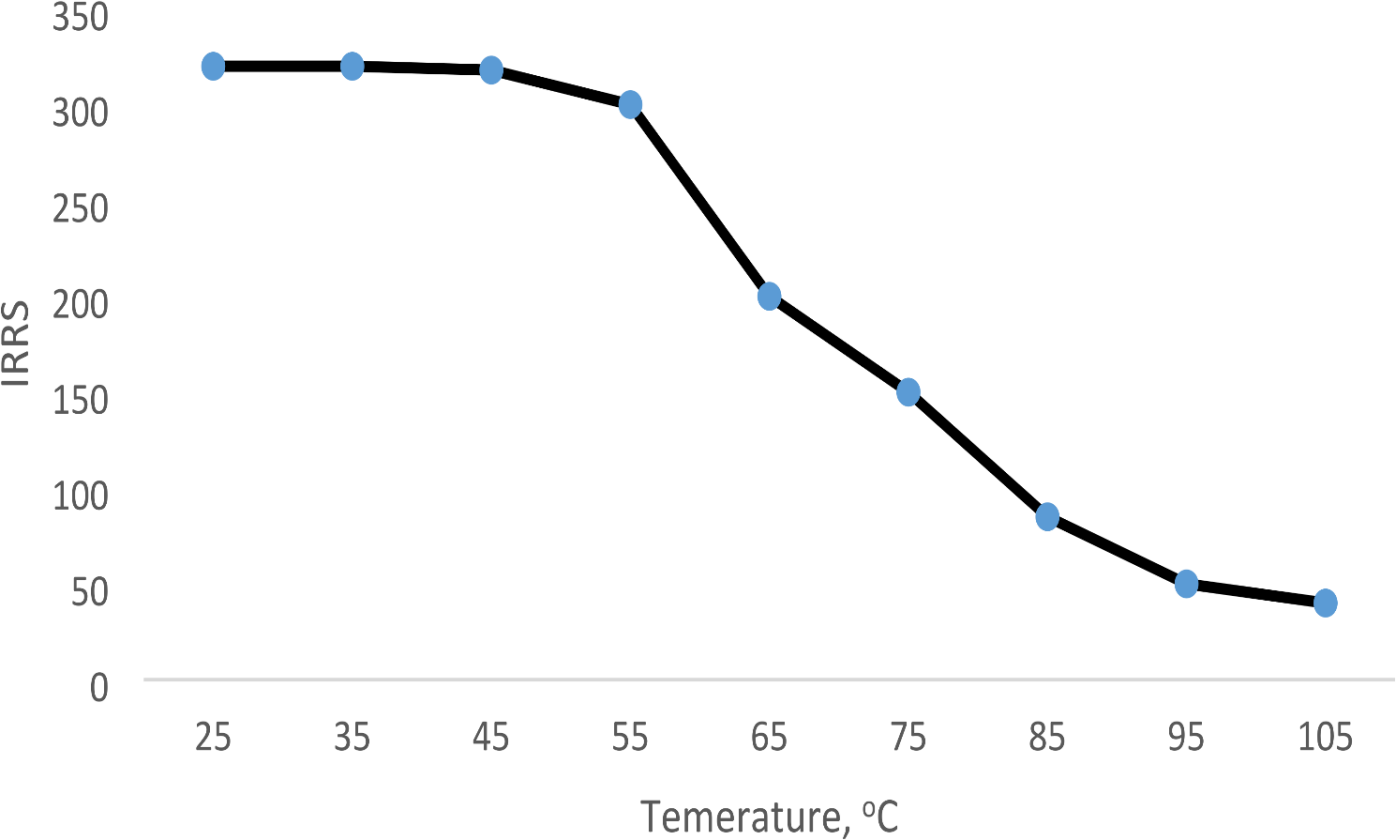
Effect of temperature on the D IRRS of the reaction product between VER and erythrosine B

The impact of temperature on the formed complex formation reaction was studied at nine different temperatures (room temperature 25, 30, 40, 50, 60, 70, 80, 90, and 100 ^o^C). Figure 7 demonstrates that the highest fluorescence values were obtained at 25–49 ^o^C. The fluorescence values were significantly decreased by increasing the temperature higher than 40 ^o^C. Therefore, the reaction was carried out at room temperature (about 25 ^o^C).

### Validation

Both methods were validated in accordance with the International Conference on Harmonization’s (ICH) Q2 requirements. To guarantee the validity of the designed sensor to be used for spectrophotometric and spectrofluorometric analysis of VER, various validation parameters were evaluated.

#### Linearity and range

**Fig. 8:**
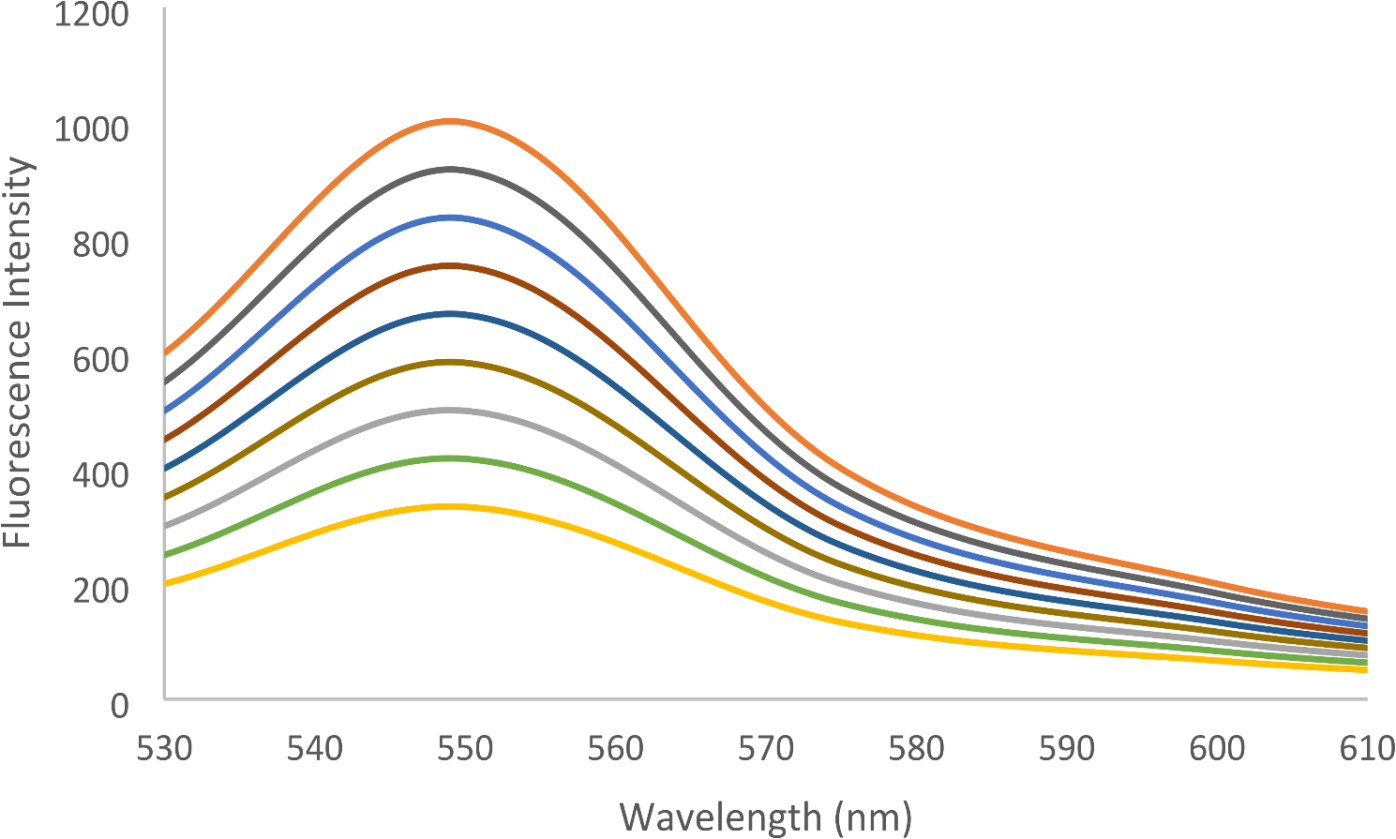
The emission spectra of the reaction products of EB with different concentrations of VER in BR buffer at pH 4

**Fig. 9:**
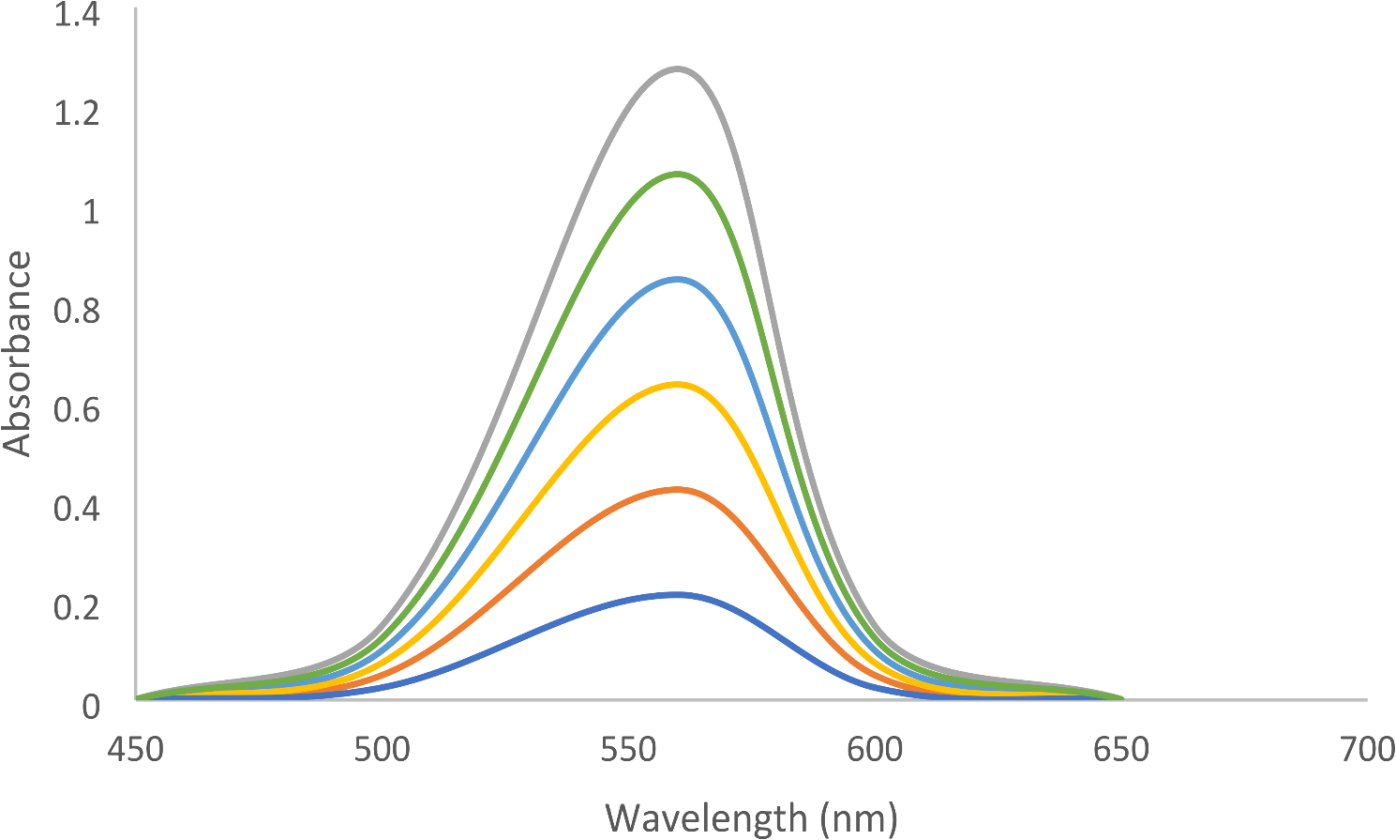
Absorption spectra of the reaction products of EB with different concentrations of VER in BR buffer at pH 4

Adding increased concentrations of VER showed a quantitative decrease in the fluorescence intensity of EB (Fig. 8). The calibration graph showed a rectilinear relationship between fluorescence quenching (ΔF) and concentrations over the range of 0.1–1.0 µg mL^− 1^ VER in the spectrofluorometric assay. Conversely, in the spectrophotometric method, increasing the concentrations of VER showed quantitative increases in the absorbance (Fig. 9). The linearity was achieved in the range of 0.5–10.0 µg mL^− 1^ VER. The linear regression parameters are shown in Table 1.

**Table 1:** Validation data of VER by the proposed methods

Parameter	Spectrofluorometric method	Spectrophotometric method
Concentration range (µg mL^− 1^)	0.05–0.5	0.5–10.0
Detection limit (µg mL^− 1^)	0.012	0.131
Quantitation limit (µg mL^− 1^)	0.036	0.412
Determination coefficient (r^2^)	0.9996	0.9999
Slope	1131	0.0578
Intercept	47.83	0.0729
S^y/x^	1.8358	0.0061
S_a_	12.467	0.0075
S_b_	17.796	0.0083
% Error	0.764	0.675
% RSD	1.964	1.432
Mean found (%) ± SD	100.76 ± 0.987	99.89 ± 1.01

Note: S_a_, standard deviation of the intercept of regression line; S_b_, standard deviation of the slope of regression line; S_y/x_, standard deviation of the residuals

#### Detection and quantitation limits

Detection and quantitation limits were calculated as guided by the ICH recommendation.

For the spectrofluorometric method, the detection limit was 0.012 µg mL^− 1^, whereas the quantitation limit was 0.036 µg mL^− 1^. For the colorimetric approach, the detection limit was found to be 0.131 µg mL^− 1^, whereas the quantitation limit was 0.412 µg mL^− 1^.

#### Precision and accuracy

Intra- and inter-day precision (repeatability and intermediate precision) of the presented approaches were confirmed via measuring three various concentrations (0.05, 0.07, and 0.09 µg mL^− 1^ for the spectrofluorometric approach and 0.5, 7.0, and 9.0 µg mL^− 1^ for the spectrophotometric approach of the analyte VER at three various times within a day (intra-day precision) and in three successive days (intermediate precision) as illustrated in Table 2. The results indicated a high percentage of found and low RSD values, confirming the high precision of the developed approaches. The methods were also compared with a comparison method to prove the accuracy of the developed methods. The used comparison method was a spectroscopic method [10]. Student’s t-test and variance ratio F-test were found to be lower than the tabulated ones [22], indicating the accuracy and precision of the developed spectrofluorometric and spectrophotometric approaches as demonstrated in Table 3.

**Table 2:** Precision data for the Estimation of VER in pure form by the proposed methods

Concentration taken (µg mL^− 1^)	% Found	% RSD
Spectrofluorometric method		
Intra-day		
0.05	99.43	1.11
0.07	98.36	1.32
0.09	100.11	1.26
Inter-day		
0.05	100.38	2.01
0.07	98.11	1.68
0.09	99.34	0.99
Spectrophotometric method		
Intra-day		
4.0	99.78	2.98
6.0	101.57	1.88
10.0	99.85	1.91
Inter-day		
5.0	99.68	1.89
7.0	98.97	0.99
9.0	98.59	1.89

**Table 3:** Application of the developed fluorometric and colorimetric methods and the comparison method for Estimation of VER in Raw materials

Parameters	Spectrofluorometric method		Comparison method (*n* = 3) [10]	Spectrophotometric method		Comparison method (*n* = 3) [10]
VER (pure form)	Amount taken (µg mL^− 1^)	% Recovery ± SD		Amount taken (µg mL^− 1^)	% Recovery ± SD	
	0.05	99.89 ± 1.11	99.76 ± 0.85	0.5	101.65 ± 0.99	100.32 ± 1.35
	0.10	100.76 ± 1.76	100.42 ± 1.01	2.5	100.98 ± 0.75	100.68 ± 1.02
	0.20	100.64 ± 0.86	99.66 ± 0.95	4.5	99.75 ± 0.54	99.99 ± 0.97
	0.30	99.07 ± 0.97		6.5	100.87 ± 1.65	
	0.40	100.26 ± 1.77		8.5	99.54 ± 1.53	
	0.50	99.75 ± 1.74		10.0	98.59 ± 1.94	
X^−^ ± SD		100.06 ± 1.37	99.95 ± 0.94		100.23 ± 1.23	100.33 ± 1.11
t -test		0.865			0.457	
F - value		1.457			1.108	

Note: Figures within parentheses are the tabulated t and F values at *p* = 0.05

Average of three separated exterminations

Theoretical values at 95% confidence interval: t = 2.776, F = 5.97

Statistical evaluation of the results of the assays was performed by one-way ANOVA test at a confidence interval (Cl) level of 95%. Results from the ANOVA test came with F-values lower than the critical value and acceptable higher P-value than the Cl level confirming no statistically significant difference between the proposed methods and the reported method as shown in Tables 3 and 4.

**Table 4:** Application of the developed spectrofluorometric and spectrophotometric methods and the comparison method for Estimation of VER in tablet form

Parameters	Spectrofluorometric method		Comparison method (*n* = 3) [10]	Spectrophotometric method		Comparison method (*n* = 3) [10]
	Amount taken (µg mL^− 1^)	% Recovery ± SD		Amount taken (µg mL^− 1^)	% Recovery ± SD	
	0.2	99.67 ± 1.15	99.76 ± 0.85	2.5	98.59 ± 0.99	100.32 ± 1.35
	0.3	100.87 ± 0.98	100.42 ± 1.01	4.5	100.97 ± 1.17	100.68 ± 1.02
	0.4	100.68 ± 1.32	99.66 ± 0.95	6.5	99.68 ± 1.87	99.99 ± 0.97
X^−^ ± SD		100.41 ± 1.15	99.95 ± 0.94		99.75 ± 1.34	100.33 ± 1.11
t -test		1.042			0.345	
F - value		1.923			0.954	

Note: Values within parentheses are the tabulated t and F values at *p* = 0.05

Average of three separated exterminations

Theoretical values at 95% confidence interval: t = 2.776, F = 5.97

#### Robustness

The effect of small variations in certain factors on the performance of the presented approaches was studied carefully. Such factors included BR buffer pH, BR buffer volume, and EB reagent volume. It was discovered that the performance of the created approaches is not significantly impacted by these minor adjustments.

### Applications

The developed method and the comparison spectrometric method [10] were employed to assay VER in A Verquvo Tablets. The results indicated that no significant variation existed between the proposed spectrometric methods and the comparison spectrometric approach when the student’s t-test and F-test were applied, as indicated in Table 4. Statistical analysis of the results produced in Table 4 revealed the excipients and additives present in the studied tablet, e.g. corn starch, crospovidone, hydroxypropyl cellulose, lactose monohydrate, magnesium stearate, mannitol, microcrystalline cellulose, and sodium lauryl sulfate, have no interference with the suggested spectrometric methods. This means the high selectivity of the produced methods.

### Reaction stoichiometry

**Fig. 10:**
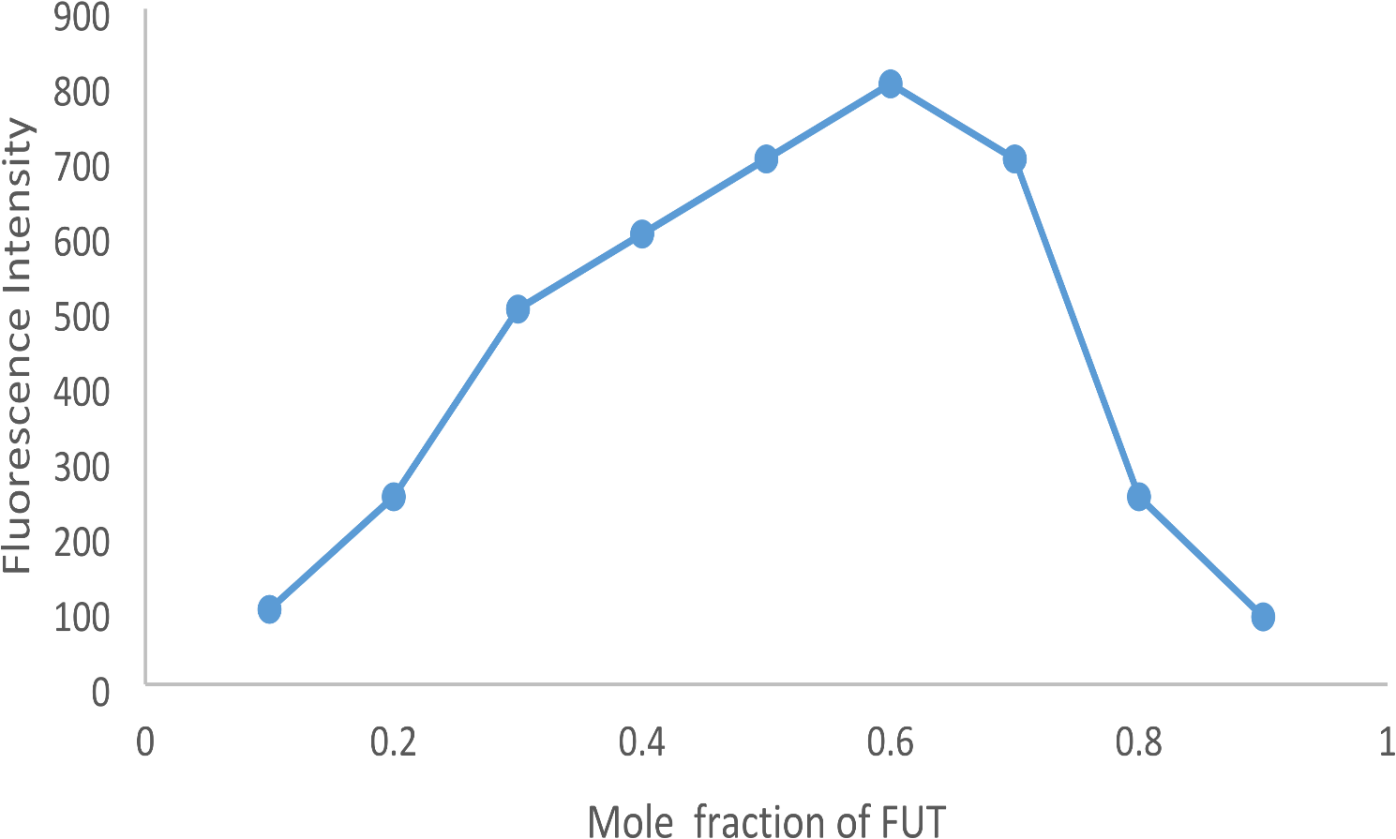
Job’s plot of the ion pair complex of VER and erythrosine Busing the same concentration of the analyte and the reagent (1 × 10 ^-4^ M)

**Scheme 1:**
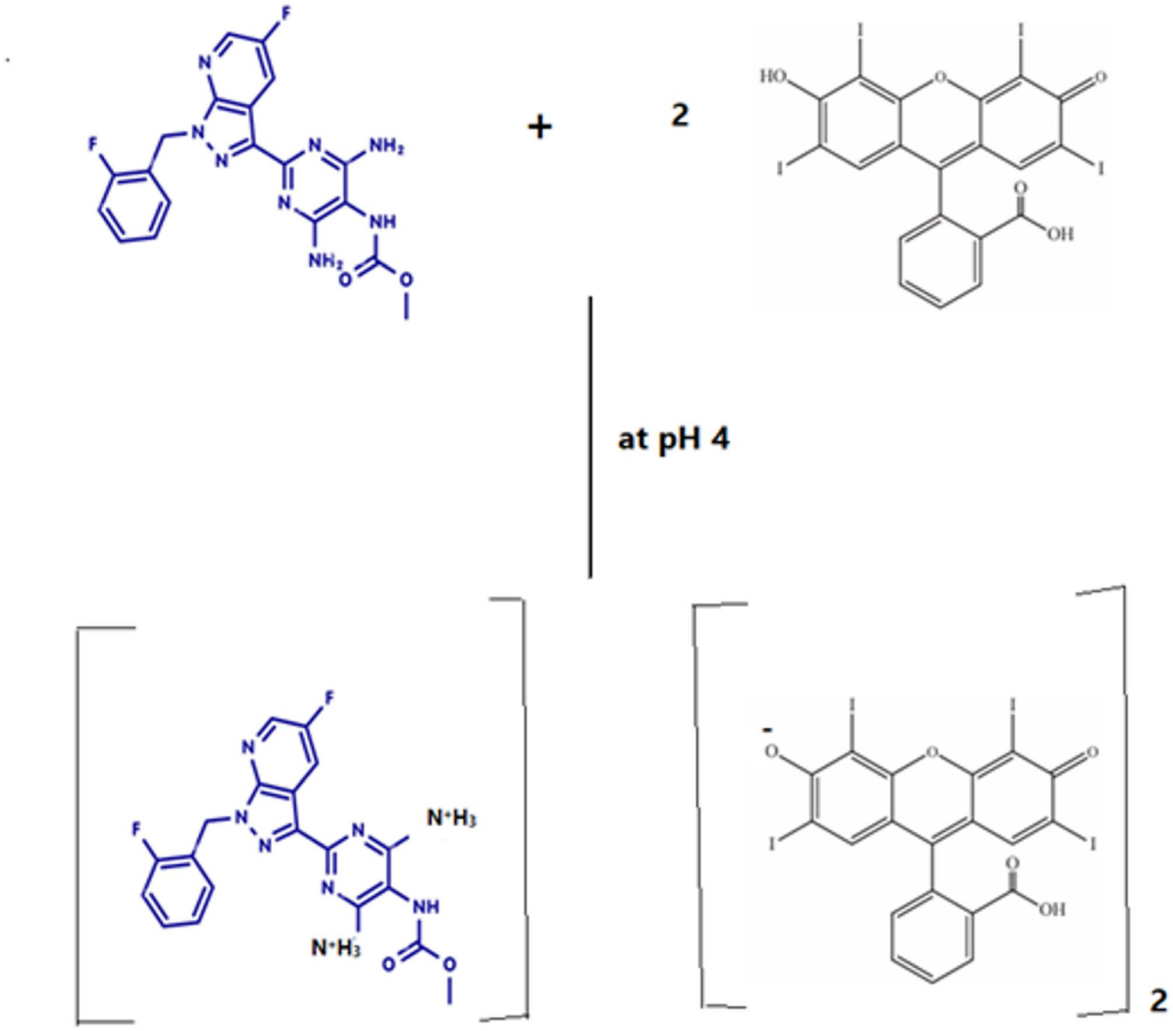
The proposed reaction mechanism for the ion-pair complex formation between VER and EB

On the mechanism of the vericiguat-erythrosine B reaction, the ratio between EB and VER in the produced ion-pair complex was estimated by applying Job’s continuous variation method. A series of solutions containing different molar ratios of the drug and the reagent with equal molar concentrations (1.7 × 10^− 4^ M) were prepared, keeping the total moles in all solutions constant. Then, the fluorescence intensities of each mixture at various mole fractions were determined, and then the ΔF was obtained by subtracting the observed fluorescence intensity from that of the solutions with the same concentration of the EB dye alone. Subsequently, the Job’s plot was constructed by graphing ΔF versus the mole fraction of VER in the mixtures, Fig. 10. The obtained curve showed a maxima point indicating that the molar ratio is (2:1) for EB: VER. The reaction mechanism between VER and EB was suggested based on the estimated molar ratio as represented in Scheme 1. In weakly acidic media (pH 4.0), EB will dissociate, producing a monovalent anion, with the OH group ionizing more than the COOH group of the benzene ring. This is because the xanthene ring has two strong electron-withdrawing iodine atoms next to the hydroxyl group. To produce the ion pair complex, the negatively charged monovalent anion of EB will engage with the drug cation via electrostatic contact as well as hydrophobic forces as illustrated in Scheme 1.

Job’s approach was utilized to estimate the reaction stoichiometry between VER and the reagent EB. The reaction stoichiometry was found to be 2:1 in both spectrofluorometric and spectrophotometric methods, as shown in Fig. 10. Erythrosine B, a xanthene dye, is a diprotic weak acid (H_2_L) having pKa_1_ of 3.9 and pKa_2_ of 5.0 in aqueous solution [23]. One acidic group of erythrosine B will be ionized to produce a monovalent anion (HL^−^ ) in a slightly acidic solution (pH 4). Even though EB has two acidic groups, phenolic and carboxylic groups, ionization of the phenolic OH group is easier than the carboxylic group owing to its location between two electron-withdrawing groups [24, 25]. The findings also show that the primary reaction is hydroxyl dissociation. The AM1 quantum chemistry method calculation findings suggest that the enthalpy alterations of hydroxyl on the xanthene group and–COOH on phenyl are 285.8 and 165.5 kJ/mol, respectively. The hydroxyl group can release more energy than that of–COOH, which of 120.3 kJ/mol. Additionally, it demonstrates that hydroxyl dissociation makes the system more stable [26]. In an acidic aqueous solution, the hydroxyl group of the erythrosine B molecule will be easily ionized, generating a monovalent anion, which bears a negative charge (HL^−^). Additionally, the positively charged cationic form of the VER molecule via two primary amino group cationization is chemically logical and simple in an acidic environment because its pKa is 13.82 [27]. Therefore, at a weakly acidic medium (pH 4), the ion-pair complex between the two primary amine-containing drugs (VER) and the reagent (EB) is formed due to the electrostatic force between the primary amino groups of VER and the phenate group of EB [28]. Various primary amine drugs have been documented to interact with EB, causing the inhibition of its inherent emission magnitude [12, 13, 27, 29].

**Fig. 11:**
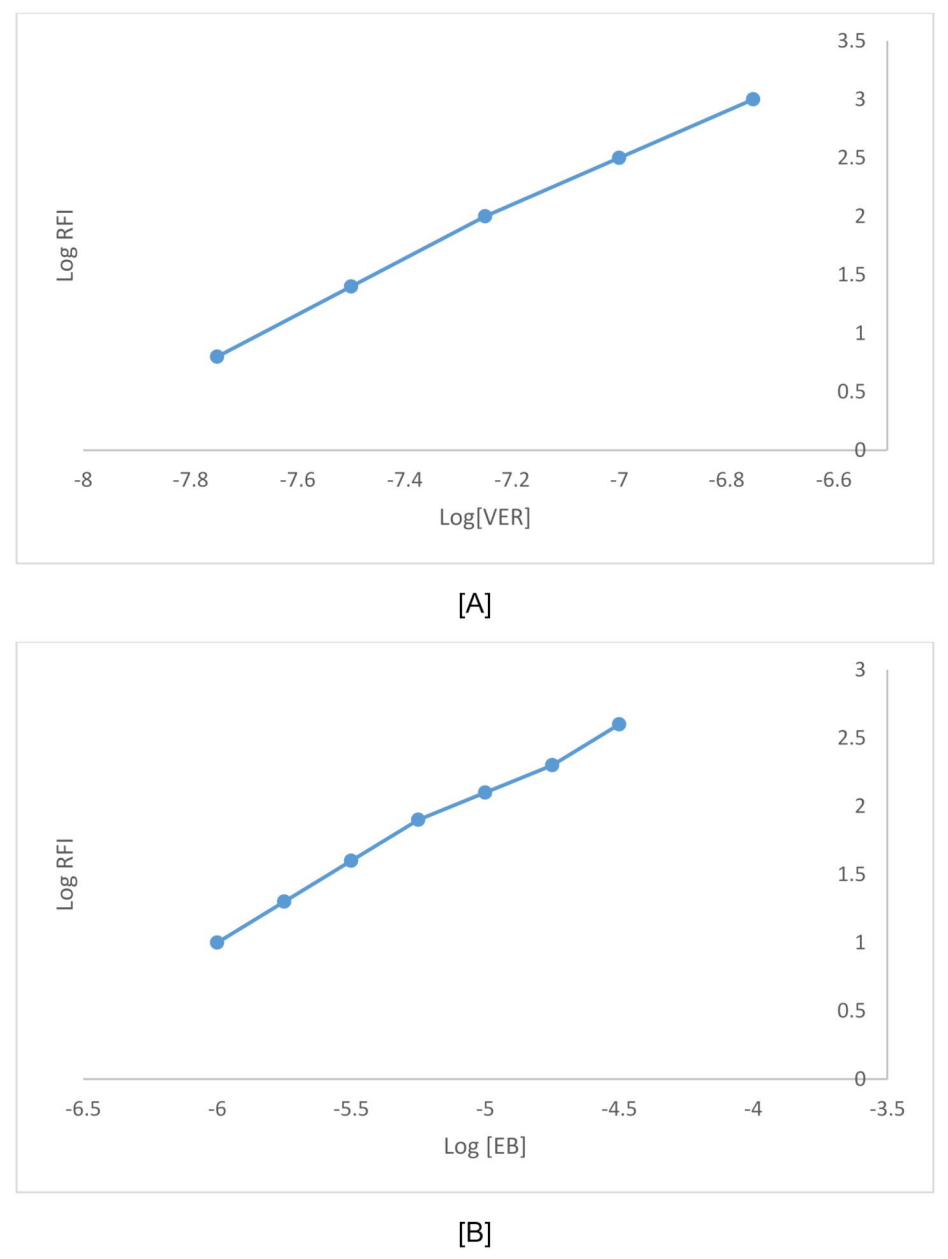
Stoichiometry of the fluorometric interaction between VER and EB using logarithmic method where **a** Log [VER] against Log RFI, **b** Log [RB] against Log RFI

Also, the reaction stoichiometry between VER and EB was studied using the limiting logarithmic method. Straight lines with slopes of 1.042/0.5214 were produced by plotting log absorbance against either log [EB] or log [VER] (Fig. 11). Thus, it is postulated that the reaction takes place in a 2:1 ratio between VER and EB (Scheme 1).

### Calculation of quantum yield

It is known that the quantum yield (Ф) of the fluorophore is a specific property that can be computed by comparison with a suitable standard [30]. In the suggested fluorophore (EB), Rhodamine 6G is selected as a reference (prepared in ethanol; reported Ф = 0.91 [31] to calculate Ф of EB.

The following equation is utilized to calculate Ф:

$${\Phi _x} = {\Phi _{st}} \times {I_x}/{I_{st}} \times {A_{st}}/{A_x} \times {n_x}/{n_{st}}$$

where Ф_x_ and Ф_st_ are the Ф of EB and Rhodamine 6G in ethanol, respectively. I is integrated fluorescence, A is the absorbance, and n is the refractive index of the solvent. The symbols (x) and (st) denote EB and the standard Rhodamine 6G, respectively.

The results revealed that EB has Ф of 4.35%, which was decreased after the addition of VER to 3.26%. This decrease in Ф assured the interaction of EB with the studied drug.

According to the charge density, electrostatic potential, and frontier orbital theories, the active sites and active bonds were obtained, and a series of reactions were given. The thermodynamic and structural parameters of each reaction were explored.

### Assessment of the greenness of the proposed methods

**Fig. 12:**
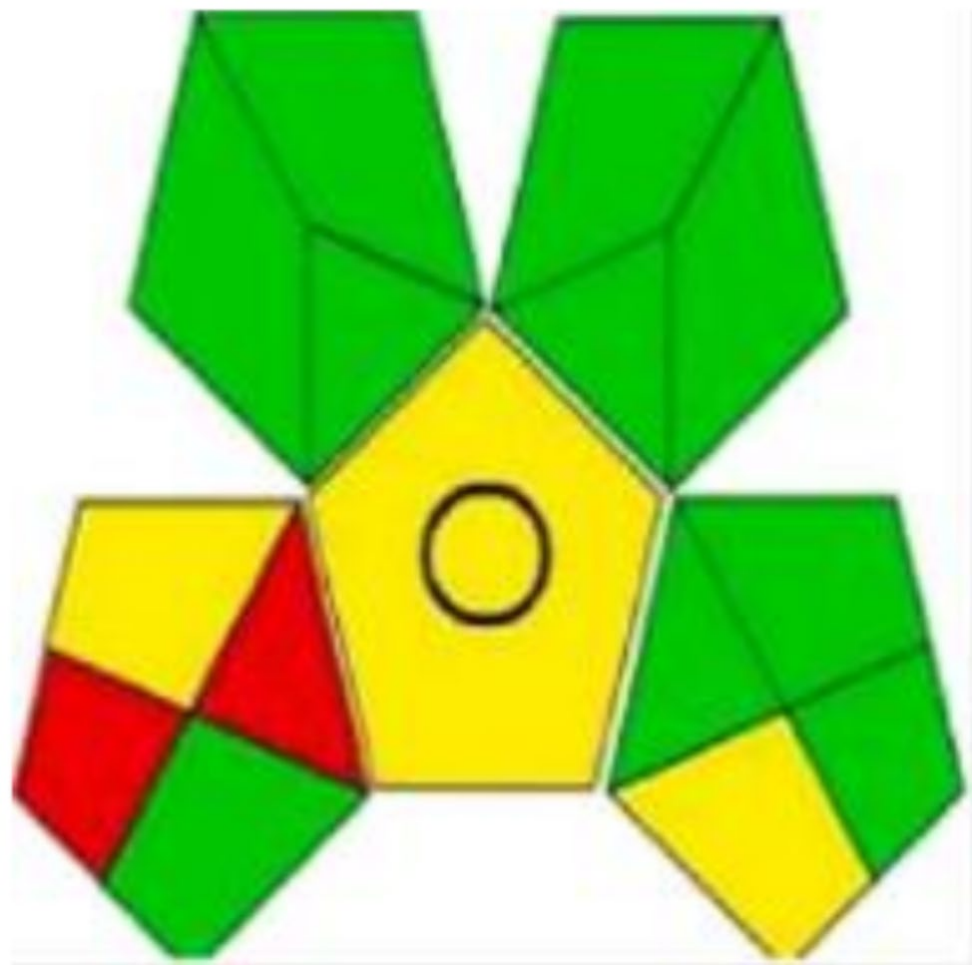
Green Analytical Procedure Index (GAPI) pictogram for the assessment of methods’ greenness

**Fig. 13:**
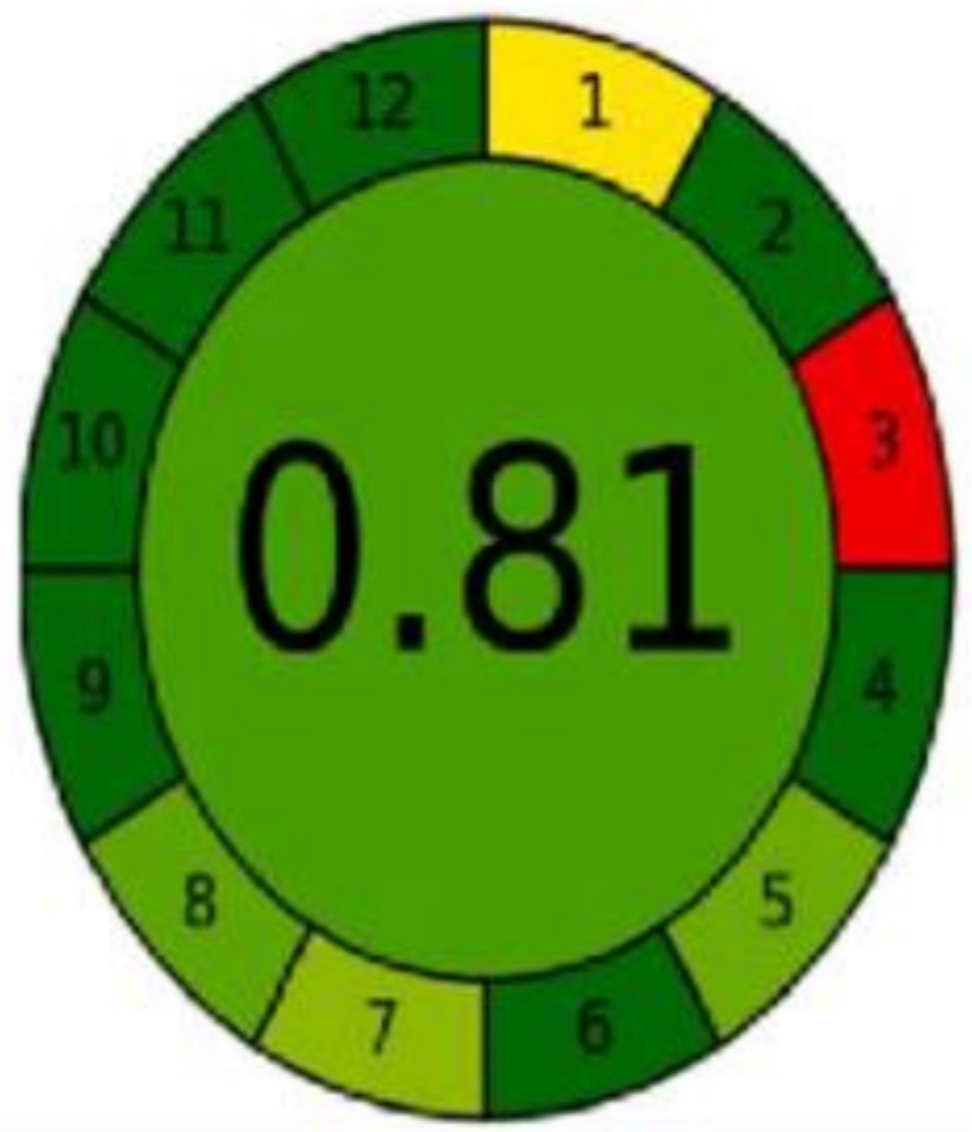
Analytical Greenness (AGREE) pictogram for the assessment of methods’ greenness

One of the main goals of green analytical chemistry (GAC) nowadays is to develop safe and environmentally friendly methods. Both the Green Analytical Procedure Index (GAPI) and Analytical Greenness (AGREE) tools have been used to assess the greenness of the.

presented methods. GAPI is used for the evaluation of the greenness of various analytical approaches employed for the analysis of pharmaceutical compounds [32–34]. It is represented by a pictogram constructed of 15 sectors; each sector has red, yellow, or red color based on the level of greenness [35]. As shown in Fig. 12, the majority of the pictogram is green. Sectors 1 and 15 are red due to offline sampling and untreated waste, respectively. Sector 14 is yellow due to the volume of 10 mL of waste. The AGREE metric was also reported for the greenness evaluation of different analytical methods and showed high efficiency [31, 33]. AGREE pictogram is a graph divided into 12 sectors representing the principles of GAC. The core of the graph has a color and score indicating the greenness score of the approach. For the developed methods, the AGREE pictogram has a green core with a score of 0.81 (Fig. 13). Both GAPI and AGREE tools prove the greenness of the proposed methods.

## Conclusion

For the first time, VER in its raw material and tablet dosage forms was analyzed using spectrofluorometric and spectrophotometric methods using the food colorant EB as a selective sensor. Using BR buffer, the ion-pair complex formed between erythrosine B and VER—which is brightly pigmented and non-fluorescent at pH = 4 is the basis for the methods that are being discussed. The spectrophotometric and spectrofluorometric approaches have linearity ranges of 0.5–10.0 µg/mL and 0.05–0.5 µg/mL, respectively, and are both simple, quick, and sensitive enough to be used for quality control analysis of VER in its pharmaceutical formulations. When compared to a previously published method, the fluorometric and colorimetric procedures showed no discernible difference and were validated in accordance with ICH requirements. The results of the statistical analysis showed sensitivity and selectivity of the suggested spectrometric methods for determination of active constituents of the studied drug in micro quantities and in the presence of corn starch, crospovidone, hydroxypropyl cellulose, lactose monohydrate, magnesium stearate, mannitol, microcrystalline cellulose, and sodium lauryl sulfate without interference. Moreover, both methods are environmentally benign as they do not incorporate hazardous organic solvents. The greenness of the presented approaches was confirmed by applying GAPI and AGREE metrics, which indicated the greenness of the presented approaches.

## Data Availability

No datasets were generated or analysed during the current study.

## References

[1] Martindale W. The complete drug reference 33d ed, Swectman, SC, editor London.Chicago. Pharmaceutical; 2002.

[2] Wenzel JP, Nikorowitsch J, bei der Kellen R, Magnussen C, Bonin-Schnabel R, Westermann D, Twerenbold R, Kirchhof P, Blankenberg S, Schrage B. Heart failure in the general population and impact of the 2021 European society of cardiology heart failure guidelines.esc heart failure, 2022. 9.2157-2169. 10.1002/ehf2.13948 PMC9288760 35445582

[3] Siddiqi AK, Greene SJ, Fudim M, Mentz RJ, Butler J, Khan MS. J.E.R.o.C.T., vericiguat for the treatment of heart failure with reduced ejection fraction. Expert Rev Cardiovasc Ther. 2023;21:245–57. 36881733 10.1080/14779072.2023.2189101

[4] Pravalika P, Jephthah G, Reddy AR, Rao TR. A NEW RP-HPLC method for Estimation of verciguat in bulk drug and pharmaceutical dosage form. J Adv Sci Res. 2023;14:37–43.

[5] Mustafa DM, Magdy N, Azab E. N.F., The first validated stability-indicating HPLC/DAD method for quantitation of vericiguat in its pharmaceutical formulation; application to degradation kinetic studies.talanta, 2023. 259124498. 10.1016/j.talanta.2023.124498 37011562

[6] Ding C, Guo C, Fang L, Li Y, Wang Z, Dong Z. Determination of vericiguat in rat plasma by UPLC-MS/MS and its application to drug interaction. J Chromatogr A, 2023. 1709.464401. 10.1016/j.chroma.2023.464401 37741219

[7] Zhang E, Chen C, Wang Y, Weng Q, Xu R-a, Lin J. An investigation of Pharmacokinetic interaction of vericiguat with apigenin based on a newly developed Ultra-performance liquid Chromatography-tandem mass spectrometry assay. J Curr Med Chem. 2024;31(9):33,. 5468–76. 10.2174/0109298673258387230921090445 37888816

[8] Mustafa DM, Magdy N, Azab E. N.F., Different spectrophotometric methods for simultaneous quantitation of vericiguat and its alkaline degradation product: a comparative study with greenness profile assessment.scientific reports, 2023. 1323077. 10.1038/s41598-023-50097-1 PMC10754859 38155184

[9] Ainousah BE, Abdelazim AH, Almrasy AA, Abdel-Kareem RF, Ghoneim MM, Gamal M. Color based spectrophotometric determination of vericiguat using Diazo coupling reaction; assisted computational calculations. J Mol Struct, 2023. 1294136317.

[10] Hesham S, Dina ZM, Bassant H, Hytham R, Alyaa A, Nourhan O, Maimana AM. Analysis of vericiguat via ion-pairing with eosin Y as a spectrofluorimetric and spectrophotometric probe: Application to content uniformity test. Spectrochimica Acta Part A: Molecular and Biomolecular Spectroscopy, 2025. 329,125482. 10.1016/j.saa.2024.125482 39631201

[11] Li Z, Sakamuru S, Huang R, Brecher M, Koetzner CA, Zhang J, Chen H, Qin C-F, Zhang Q-Y, Zhou J, Kramer LD, Xia M, Li H. Erythrosin B is a potent and broad-spectrum orthosteric inhibitor of the flavivirus NS2B-NS3 protease J.Anti viral res. (2018). 10.1016/j.antiviral.2017.12.018 PMC5892443 29288700

[12] Hamad A, Ali R, Ali HRH, Nagy DM, Derayea SM. Facile complexation reactions for the selective spectrofluorimetric determination of albendazole in oral dosage forms and spiked human plasma J.RSC Adv.(2018). 10.1039/c7ra12360d PMC9078121 35542411

[13] Derayea SSM, Hamad AA, Ali R. H. R. H. Ali,Investigating erythrosine B as a fluorimetric probe for the determination of benzimidazole drugs via facile complexation reaction. J Microchemical J(2019).

[14] Ibrahim F, Aboshabana R, Elmansi H. Spectroscopic strategies for quantitation of varenicline in pharmaceutical preparations and content uniformity testing. J R Soc Open Sci (2022). 10.1098/rsos.220628 PMC9515633 36177195

[15] Yosrey E, Elmansi H, Sheribah ZA, Metwally MES. Novel approaches of erythrosine B as a food dye-derived spectroscopic probe for assessing trospium chloride in Raw material and dosage form.J.Luminescence.(2022). 10.1002/bio.4358 35922904

[16] Tolba M, Salim M. Insights for applying erythrosine B as a green fluorescence probe for Estimation of anticancer Tamoxifen and its analog; clomiphene in nanogram concentration J.Spectrochimica acta part A. Molecular and Biomolecular Spectroscopy.; 2021. 10.1016/j.saa.2021.120156 34293668

[17] El Sharkasy ME, Tolba MM, Belal F, Walash MI. R. AboShabana,Utility of the food colorant erythrosine B as an effective green probe for quantitation of the anticancer Sunitinib. Application to pharmaceutical formulations and human plasma.J. Luminescence; 2023. 10.1002/bio.4598 37747151

[18] Almahri A, Abdel-Lateef MA, Samir E, Derayea SM. M. A. El Hamd, resonance Rayleigh scattering and spectrofluorimetric approaches for the selective determination of rupatadine using erythrosin B as a probe: application to content uniformity testJ.Luminescence.(2021). 10.1002/bio.3983 33179860

[19] Binkadem MS, AlSalem HS, Al-Goul ST, El Hamd MA, Oraby M, Ali Zainy FM. M. A. Abdel-Lateef, validated spectrofluorimetric and resonance Rayleigh scattering methods for determining Naftidrofuryl in varied pharmaceutical samples based on its interaction with erythrosin B,J.Luminescence.(2023). 10.1002/bio.4570 37555794

[20] Płotka-Wasylka J. A new tool for the evaluation of the analytical procedure. Green Analytical Procedure Index,J.Talanta; 2018. 10.1016/j.talanta.2018.01.013 29426502

[21] Pena-Pereira F, Wojnowski W. M. Tobiszewski AGREE—Analytical greenness metric approach and software. J Anal Chem. (2020). 10.1021/acs.analchem.0c01887 PMC7588019 32538619

[22] Miller J. J. C. Miller,Statistics and chemometrics for analytical chemistry. Harlow, England: Pearson Education; 2018.

[23] Vodolazkaya NA, Gurina YA, Salamanova NV, McHedlov NO, Petrossyan. Spectroscopic study of acid–base ionization and tautomerism of fluorescein dyes in direct microemulsions at high bulk ionic strength. J Mol Liq. 2009.

[24] Wang J, Liu Z, Liu J, Liu S, Shen W. Study on the interaction between fluoroquinolones and erythrosine by absorption, fluorescence and resonance Rayleigh scattering spectra and their application. J Spectrochim Acta Mol Biomol Spectrosc.(2008). 10.1016/j.saa.2007.05.057 17618827

[25] McHedlov-Petrossyan NO. Mayorga,Extraordinary character of the solvent influence on protolytic equilibria: inversion of the fluorescein ionization constants in H < sub > 2 O–DMSO mixtures. J Chem Soc FaradayTrans. 1992;88:3025.

[26] Tang X, Liu Z, Liu S. X. Hu,study on the interaction between diphenydramine and erythrosin by absorption,fluorescence and resonance Rayleigh scattering Spectram,J.science in China seriesB:Chemistry.(2007).

[27] 26, Moffat AC, Osselton MD, Widdop B, Watts J. Clarke’s analysis of drugs and poisons. London, UK: Pharmaceutical; 2011.

[28] Li C, Liu S, Liu Z. X. Hu, study on the interaction between verapamil hydrochloride and Eosin y by absorption,fluorescence and resonance Rayleigh scattering spectra and their analytical application. J Fluoresc. (2011). 10.1007/s10895-010-0762-6 20978828

[29] Abdel-Lateef MA, Derayea SM, El-Deen DAMN, Almahri A, Oraby M. Investigating the interaction of terbinafine with Xanthenes dye for its feasible determination applying the resonance Rayleigh scattering method,j. R Soc Open Sci. (2021). 10.1098/rsos.201545 PMC7890510 33614086

[30] Würth C, Grabolle M, Pauli J. Relative and absolute determination of fluorescence quantum yields of transparent samples,Nat. Protoc. (2013). 10.1038/nprot.2013.087 23868072

[31] Zeid AM, El-Masry AA, El-Wasseef DR, Eid M. I. A. Shehata,Green microemulsion electrokinetic chromatographic method for simultaneous determination of azelastine and Budesonide,Chem. Pharm (2022).

[32] Aboshabana R, Zeid AM. F. A. Ibrahim,Label-free green Estimation of Atenolol and ivabradine hydrochloride in pharmaceutical and biological matrices by synchronous spectrofluorimetry,j.spectrochim. Acta A mol. Biomol Spectrosc. (2023). 10.1016/j.saa.2023.122626 36940537

[33] El-Masry AA, Zeid AM. Acriflavine: an efficient green fluorescent probe for sensitive analysis of aceclofenac in pharmaceutical formulations,j. BMC Chem. (2023). 10.1186/s13065-023-00979-2 PMC10394782 37533016

[34] El-Masry AA. A. M. Zeid,Nano-scale analytical insights for determination of Vonoprazan and aspirin in a recently approved combined Preparation utilizing nucleophilic substitution reaction, along with evaluation approaches for both greenness and Whiteness,J. Microchem. (2024).

[35] Imam MS, Abdelazim AH, Ramzy S, Batubara AS, Gamal M, Abdelhafiz S. A. M. Zeid,Adjusted green spectrophotometric determination of favipiravir and Remdesivir in pharmaceutical form and spiked human plasma sample using diferent chemometric supported models. J BMC Chem. (2023). 10.1186/s13065-023-01001-5 PMC10373238 37501208

